# Predictors of unsuppressed viral load among MSM with HIV in South Africa

**DOI:** 10.1097/QAD.0000000000004205

**Published:** 2025-05-21

**Authors:** Jessica S. van der Mannen, Stanford Furamera, Jacqueline Pienaar, Pontsho Komane, Luxolo Shokota, Boitumelo Ramashala, Lindiwe Tsope, Elise M. van der Elst, Danielle Giovenco, Don Operario, Eduard J. Sanders

**Affiliations:** a Implementation Research Division, The Aurum Institute, Johannesburg, South Africa; b Division of Infectious Diseases & Public Health, Department of Health Sciences, Vrije Universiteit Amsterdam, Amsterdam, The Netherlands; c Health Systems Division, The Aurum Institute, Johannesburg, South Africa; d Department of Interdisciplinary Social Science, Utrecht University, Utrecht, The Netherlands; e Behavior, Social and Health Education Sciences, Emory University, Atlanta, Georgia, USA; f Department of Global Health, University of Amsterdam, Amsterdam, The Netherlands; g Dunn School of Pathology, University of Oxford, Oxford, UK.

**Keywords:** adherence, anxiety, antiretroviral therapy, HIV, medication adherence, mental health, MSM, South Africa, unsuppressed viral load

## Abstract

**Introduction:**

South Africa has the largest HIV epidemic of the world, but predictors of unsuppressed viral load among MSM are scarce.

**Methods:**

We did a cross-sectional study to assess socio-demographic characteristics, year of antiretroviral therapy (ART) initiation, and viral load data from 1385 MSM registered in five key population clinics in five districts in South Africa in 2023 and 2024. We used logistic regression to assess predictors of unsuppressed viral load (≥200 copies/ml). We then conducted a case--control study involving 57 cases (viral load≥200 copies/ml) and 57 matched controls (viral load<200 copies/ml; matched on age, ART start date, clinic, and outreach vs. facility attendance) drawn from the cross-sectional study to explore additional differences between groups.

**Results:**

In the cross-sectional study, predictors for unsuppressed viral load included on ART for 1–2 years [odds ratio (OR) 3.53; 1.77–7.69] or 3–5 years (OR 2.33; 1.18–5.04) vs. more than 5 years, being an outreach vs. facility-based client (OR: 2.64; 1.79–3.94), and enrolled at Johannesburg (OR: 3.53; 2.25–5.64), or Durban (OR: 0.36; 0.84–0.70), vs. Pretoria. The case–control study identified predictors for unsuppressed viral load, which included missing more than four ART dosages per month (OR: 5.55; 1.19–41,03), having moderate to severe anxiety (OR: 3.90; 1.34–12,52), hazardous alcohol use (OR: 2.70; 1.14–6.81), and Christian vs. no religion (OR: 3.89; 1.34–12.29).

**Conclusion:**

We identified regional differences and key predictors of unsuppressed viral load among MSM living with HIV in South Africa. Screening for risk factors for unsuppressed viral load at ART continuation may inform where adherence support and other health services are needed.

## Introduction

South Africa has the largest HIV epidemic globally, with around 8 million people living with HIV and approximately 150 000 new infections annually [1–3]. The Joint United Nations Programme on HIV/AIDS (UNAIDS) introduced the 95-95-95 targets to control the epidemic by 2030, aiming for 95% of people with HIV (PWH) to know their status, 95% of those diagnosed with HIV to be on antiretroviral therapy (ART), and 95% of those on ART to achieve viral suppression [4].

Men who have sex with men (MSM) face high HIV transmission risks and challenges in care engagement [5,6]. A 2022 systematic review and meta-analysis found that South African MSM have 2.7 times the HIV prevalence than the general male population, with an estimated prevalence of 30–36% [7]. In Johannesburg, the estimated proportion of MSM on ART and who were virally suppressed was 47% in 2019 [8].

Poor ART adherence, largely due to social, psychological, and behavioral barriers, is a key contributor to unsuppressed viral load [9]. Further, stigma, discrimination, and nondisclosure of sexual orientation and HIV-status may hinder access to care among South African MSM [10–12]. Common mental health challenges such as depression and anxiety may present medication adherence challenges [13–15]. A key population services program (“POP INN” clinics) has been established in South Africa in response to care cascade challenges among MSM. This study assessed predictors of unsuppressed viral load among MSM enrolled in the five POP INN clinics in South Africa.

## Methodology

### Study setting and participants

The Aurum POP INN key population program offers HIV prevention and treatment to over 20 000 individuals in South Africa, including MSM. POP INN clients receive free PrEP or ART, adherence counseling, HIV tests every 3 months for those on PrEP, and once-yearly viral load testing for those on ART more than 1 year.

#### Cross-sectional study

Data for the cross-sectional study derived from a services database maintained at each of the five POP INN clinics: Ehlanzeni (Nelspruit), Ekurhuleni (Johannesburg), eThekwini (Durban), Tshwane (Pretoria), and uMgungundlovu (Pietermaritzburg). Clients who identified as cisgender MSM, initiated ART (dolutegravir/lamivudine/tenofovir regimen) more than 1 year prior, and had a viral load result in the period January 2023–April 2024 were included in this study.

#### Measures

Measures included POP INN clinic location, client group (i.e., ART collected at facility/or provided during outreach), date of birth, ART initiation date, initiated ART at the POP INN clinic or elsewhere, and if client has previously been on ART. Only the client's most recent viral load was included and dichotomized at least 200 copies/ml. Participants with viral load less than 200 copies per ml were considered to have suppressed viral load.

#### Case–control study

A case–control study was conducted in Tshwane and Ekurhuleni in the period 30 April–17 July 2024, with cases deriving from the cross-sectional study. Cases were identified based on clients whose most recent viral load result were at least 200 HIV copies/ml. Cases had a median viral load of 1010 (IQR: 426–32 500) copies/ml. For each case, a control was matched based on age (± 2 years), date of ART initiation (± 12 months), client group (facility/outreach), and POP INN clinic location. Selected cases and controls were invited to attend a scheduled facility visit or were purposefully contacted during outreach to participate in the case-control study. Enrolled participants were reimbursed with 400 ZAR (∼20 Euro) for their participation.

Participants completed a computer-administered survey in English that assessed socio-demographics, sexual behaviors, ART adherence [16], experiences of abuse [17], depressive symptoms [Patient Health Questionnaire 9 (PHQ-9)] [18,19], generalized anxiety disorder (GAD-7) [20], alcohol use [Alcohol Use Disorder Identification Test (AUDIT-C)] [21], use of substances other than alcohol and tobacco [Drug Abuse Screening Test 10 (DAST-10)] [22], sexual stigma (abridged China MSM Stigma Scale) [23,24], and HIV-stigma (12-item short-version Berger HIV Stigma scale) [25,26]. Details on measures are provided in Appendix 1. Participants who indicated challenges reading English were assisted during the questionnaire.

### Statistical analysis

#### Cross-sectional study

We used multivariable logistic regression to assess predictors of unsuppressed viral load. Ten clients were excluded from the analysis due to missing data. The continuous variables “Years on ART” and “Days since VL” were categorized to better fit the data. Variables with more than two outcome categories (“POP INN clinic,” “Years on ART” and “Days since last VL”) were modeled as dummy variables. A backward stepwise selection (*P* < 0.05) was employed to identify predictors of unsuppressed viral load.

#### Case–control study

We used unconditional logistic regression to assess predictors of unsuppressed viral load in the case–control study [27]. The continuous variables “Years lived in Gauteng” (0–2, 3–15, >15), “Days forgotten ART in last month” (0–4, >4), and “Number of people disclosed ART-use to” (0–2, 3–5, >5) were categorized to better fit the data. Variables with more than two categories were modelled as dummy variables (e.g., religion, marital status, education).

The associations of the most important predictors for unsuppressed viral load were assessed by employing a two-step procedure to develop the prediction model. First, the most strongly associated predictors were preselected using univariable logistic regression. Only variables with a *P* value 0.15 or less were included in the final model. Subsequently, the final model was created from the set of preselected variables and validated using backward stepwise selection (*P* < 0.05). Variables with a *P* value less than 0.05 were interpreted to be associated with unsuppressed viral load.

Statistical analyses were performed using RStudio statistical software version 4.2.2.

The study was ethically approved by the Human Research Ethics Committee at the University of the Witwatersrand (AUR2-19-422).

## Results

### Cross-sectional study

Data derived from 1385 MSM living with HIV, registered at one of the five POP INN clinics, and with a viral load result during January 2023–April 2024 (Table 1). The median age of the participants was 36 years (IQR: 31–42) and 892 clients (64.4%) were outreach clients. The largest proportion of participants was from Tshwane clinic (32.5%), while the smallest proportion of participants was from Ehlanzeni clinic (11.3%). The majority (51.3%) of the participants were on ART for 3–5 years [median: 2.5, IQR: 1.6–3.7)].

**Table 1:** Characteristics of cross-sectional study population and risk factors for unsuppressed viral load in men who have sex with men registered for HIV ART care at five POP INN clinics, South Africa, 2024.

Characteristics	Overall (*N* = 1385) *n* (% all)	VL ≥ 200 copies/ml *n* (% group)	Odds ratio	95% confidence interval	*P*
POP INN clinic, *n* (%)					**< 0.001**
Ehlanzeni – Nelspruit	157 (11.3)	29 (14.4)	1.16	0.67–2.00	0.585
Ekurhuleni – Johannesburg	332 (24.0)	82 (40.8)	**3.53**	**2.25–5.64**	**< 0.001**
eThekwini - Durban	224 (16.2)	12 (6.0)	**0.36**	**0.18–0.70**	**0.004**
Tshwane - Pretoria	450 (32.5)	44 (21.9)	^∗^	^∗^	^∗^
uMgungundlovu - Pietermaritzburg	222 (16.0)	34 (16.9)	1.26	0.75–2.09	0.379
*All*		*201 (14.5)*			
Client group, *n* (%)					**< 0.001**
Facility client	493 (36.6)	53 (26.4)	^∗^	^∗^	^∗^
Outreach client	892 (64.4)	148 (73.6)	**2.64**	**1.79–3.94**	**< 0.001**
Age in years, median (IQR)	36 (31–42)	36 (31–42)	NI		
Years on ART, *n* (%)					**< 0.001**
1–2	472 (34.1)	87 (43.3)	**3.53**	**1.77–7.69**	**< 0.001**
3–5	711 (51.3)	103 (51.2)	**2.33**	**1.18–5.04**	**0.021**
> 5	202 (14.6)	11 (5.5)	^∗^	^∗^	^∗^
Days since last VL, *n* (%)					
0–90	230 (16.6)	37 (18.4)	NI		
91–180	371 (26.8)	55 (27.4)	NI		
> 180	784 (56.6)	109 (54.2)	NI		
Transferred-in, *n* (%)					
No	947 (68.4)	162 (80.6)	NI		
Yes	438 (31.6)	39 (19.4)	NI		
Initiated on ART before, *n* (%)					
No	923 (66.6)	162 (80.6)	NI		
Yes	462 (33.4)	39 (19.4)	NI		

ART, antiretroviral therapy; SD, standard deviation; VL, viral load; VLS, viral load suppression, characterized by a viral load ≥ 200 copies/ml. NI, not included in the final model.

∗ Reference category. *P* values of statistically significant predictors (*P* < 0.05).

There was a total of 201 (14.5%) cases with a viral load at least 200 copies/ml. POP INN clinic Ekurhuleni had the highest proportion of unsuppressed viral load (24.7%), followed by Ehlanzeni (18.5%), uMgungundlovu (15.3%), Tshwane (9.8%), and eThekwini (5,4%).

POP INN clinic location, client group, and years on ART were included in the final prediction model. Being enrolled in the POP INN clinic Ekurhuleni was the most important predictor [odds ratio (OR) 3.53, 95% confidence interval (95% CI) 2.25–5.64, *P* < 0.001]. Other significant variables in the prediction model were POP INN clinic eThekwini (OR = 0.36, 95% CI 0.18–0.70, *P* < 0.004), being an outreach client (OR = 2.64, 95% CI 1.79–3.94, *P* < 0.001), being 1–2 years (vs. >5 years) on ART (OR = 3.53, 95% CI 1.77–7.69, *P* < 0.001), and 3–5 years on ART (OR = 2.33, 95% CI 1.18–5.04, *P* = 0.021), Table 1.

### Case–control study

Twenty-nine cases and 29 controls from Ekurhuleni and 28 cases and 28 controls from Tshwane were enrolled. Table 2 summarizes characteristics of participants from the case–control study and presents predictors of unsuppressed viral load. The mean age of the cases was 36.6 years (IQR: 31.5–41.6) and for the controls 35.4 years (IQR: 30.2–40.8). The majority of cases and controls were outreach clients (both 71.9%). Approximately one-fifth (17.5%) of the cases indicated missing more than four ART dosages in the last month, compared to 3.5% of the controls. Moderate to severe anxiety and problematic substance abuse were more prevalent among cases (28.1 and 38.6%, respectively) than in controls (12.3 and 24.6%, respectively). The prevalence of experiences of abuse and hazardous drinking behavior was high in both cases (56.1 and 59.6%, respectively) and controls (49.1 and 45.6%, respectively).

**Table 2:** Characteristics of the case–control study population and predictors of unsuppressed viral load in MSM in Ekurhuleni and Tshwane POP INN clinics, South Africa, 2024.

	Characteristics study population		Univariable logistic regression parameters			Final prediction model parameters		
Predictor	Controls (*N* = 57)	Cases (*N* = 57)	Odds ratio	95% confidence interval	*P*	Odds ratio	95% confidence interval	*P*
Sociodemographic information								
POP INN Site, *n* (%)								
Ekurhuleni	29 (50.9)	29 (50.9)	^∗^	^∗^	^∗^			
Tshwane	28 (49.1)	28 (49.1)	1.00	0.48–2.09	1.00			
Age in years, median (IQR)	35.4 (30.2–40.8)	36.6 (31.5–41.6)	1.01	0.97–1.07	0.580			
Client group, *n* (%)								
Facility client	16 (28.1)	16 (28.1)	^∗^	^∗^	^∗^			
Outreach client	41 (71.9)	41 (71.9)	1.00	0.44–2.27	1.00			
Years on ART, median (IQR)	3 (2–4)	2 (1–3)	0.97	0.77–1.22	0.795			
Identified sex, *n* (%)								
Male	56 (98.2)	56 (98.2)	^∗^	^∗^	^∗^			
Other	1 (1.8)	1 (1.8)	1.00	0.04–25.71	1.00			
Education, *n* (%)								
Grade 1–7 (Primary school)	3 (5.3)	3 (5.3)	^∗^	^∗^	^∗^			
Grade 8–12 (High school)	30 (52.6)	37 (64.9)	1.23	0.21–7.09	0.806			
Tertiary education from a TVET	4 (7.0)	5 (8.8)	1.25	0.15–10.59	0.833			
Tertiary education (college or university)	20 (35.1)	12 (21.1)	0.60	0.10–3.70	0.568			
Current student, *n* (%)								
No	52 (91.2)	51 (89.5)	^∗^	^∗^	^∗^			
Yes	5 (8.8)	6 (10.5)	1.22	0.35–4.49	0.751			
Ethnicity, *n* (%)								
South African	52 (91.2)	52 (91.2)	^∗^	^∗^	^∗^			
Other	5 (8.8)	5 (8.8)	1.00	0.26–3.79	1.000			
Race, *n* (%)								
Black	52 (91.2)	51 (89.5)	^∗^	^∗^	^∗^	NI	NI	NI
Other	5 (8.8)	6 (10.5)	0.19	0.01–1.20	0.130	NI	NI	NI
Years lived in Gauteng, *n* (%)								
0–2	4 (7.0)	0 (0.0)	^∗^	^∗^	^∗^			
3–15	16 (28.1)	12 (21.1)	3.17e7	0.00-N/A	0.989			
> 15	37 (64.9)	45 (78.9)	1.90e7	0.00-N/A	0.989			
Religion, *n* (%)								**0.016**
No religion	16 (28.1)	11 (19.3)	^∗^	^∗^	^∗^	^∗^	^∗^	^∗^
Christian	21 (36.8)	35 (61.4)	2.42	0.96–6.34	0.065	**3.89**	**1.34–12.29**	**0.015**
Indigenous African Church	19 (33.3)	9 (15.8)	0.69	0.22–2.07	0.508	0.86	0.26–2.84	0.798
Other	1 (1.8)	2 (3.5)	2.91	0.25–67.27	0.406	5.79	0.36–159.14	0.220
Marital status, *n* (%)								
Married	4 (7.0)	4 (7.0)	^∗^	^∗^	^∗^			
Divorced	6 (10.5)	1 (1.8)	0.17	0.01–1.67	0.165			
Single	41 (71.9)	48 (84.2)	1.17	0.26–5.23	0.831			
Separated	2 (3.5)	3 (5.3)	1.50	0.16–16.80	0.725			
Co-habiting	4 (7.0)	1 (1.8)	0.25	0.01–2.75	0.295			
Current living situation, *n* (%)								
Alone	23 (40.4)	32 (56.1)	^∗^	^∗^	^∗^	NI	NI	NI
With family	22 (38.6)	19 (33.3)	0.62	0.27–1.40	0.251	NI	NI	NI
With partner	12 (21.1)	6 (10.5)	0.36	0.11–1.07	0.073	NI	NI	NI
Employment, *n* (%)								
Unemployed	31 (54.4)	36 (63.2)	^∗^	^∗^	^∗^			
Employed full-time	14 (24.6)	15 (26.3)	0.92	0.38–2.22	0.856			
Employed part-time	4 (7.0)	2 (3.5)	0.43	0.06–2.36	0.349			
Self-employed	6 (10.5)	4 (7.0)	0.57	0.14–2.19	0.421			
Between jobs (casual)	2 (3.5)	0 (0.0)	0.00	N/A-1.98e63	0.988			
Sexual behavior in past 3 months								
Sexual partner, *n* (%)								
Male	35 (61.4)	40 (70.1)	^∗^	^∗^	^∗^			
Female	16 (28.1)	11 (19.3)	0.62	0.25–1.50	0.289			
Don’t know	0 (0.0)	1 (1.8)	5.17e6	0.00-N/A	0.992			
Both	6 (10.5)	5 (8.8)	0.75	0.20–2.69	0.654			
Been paid for sex, *n* (%)								
No	48 (84.2)	49 (86.0)	^∗^	^∗^	^∗^			
Yes	9 (15.8)	8 (14.0)	0.87	0.30–2.46	0.793			
Have paid for sex, *n* (%)								
No	48 (84.2)	47 (82.5)	^∗^	^∗^	^∗^			
Yes	9 (15.8)	10 (17.5)	1.13	0.42–3.10	0.802			
Participated in group sex, *n* (%)								
No	48 (84.2)	49 (86.0)	^∗^	^∗^	^∗^			
Yes	9 (15.8)	8 (14.0)	0.87	0.30–2.46	0.793			
ART adherence								
Attitude toward taking ART pills, *n* (%)								
I take it exactly as prescribed	53 (93.0)	47 (82.5)	^∗^	^∗^	^∗^	NI	NI	NI
I take it less than prescribed	4 (7.0)	10 (17.5)	2.82	0.88–10.83	0.097	NI	NI	NI
Forget taking ART, *n* (%)								
No	43 (75.4)	30 (52.6)	^∗^	^∗^	^∗^	NI	NI	NI
Yes	14 (24.6)	27 (47.4)	2.76	1.26–6.26	0.012	NI	NI	NI
Missed days of ART in last month, *n* (%)								**0.048**
0–4	55 (96.5)	47 (82.5)	^∗^	^∗^	^∗^	^∗^	^∗^	^∗^
> 4	2 (3.5)	10 (17.5)	5.85	1.45–39.32	0.027	**5.55**	**1.19–41.03**	**0.048**
Disclosure number, *n* (%)								
0–2	18 (31.6)	21 (36.8)	^∗^	^∗^	^∗^			
3–5	13 (22.8)	13 (22.8)	0.86	0.31–2.32	0.761			
> 5	26 (45.6)	23 (40.4)	0.76	0.32–1.76	0.520			
Mental health challenges								
Experienced abuse, *n* (%)								
No (<1)	29 (51.9)	25 (43.9)	^∗^	^∗^	^∗^			
Yes (≥1)	28 (49.1)	32 (56.1)	1.33	0.64–2.79	0.453			
Depressive symptoms (PHQ-9), *n* (%)								
Minimal to mild (0–9)	44 (77.2)	40 (70.2)	^∗^	^∗^	^∗^			
Moderate to severe (10–27)	13 (22.8)	17 (29.8)	1.44	0.62–3.38	0.396			
Anxiety (GAD-7), *n* (%)								**0.016**
Minimal to mild (0–9)	50 (87.7)	41 (71.9)	^∗^	^∗^	^∗^	^∗^	^∗^	^∗^
Moderate to severe (10–27)	7 (12.3)	16 (28.1)	2.79	1.08–7.85	0.040	**3.90**	**1.34–12.52**	**0.016**
Hazardous alcohol use (AUDIT-C), *n* (%)								**0.029**
Low (0–4)	31 (54.4)	23 (40.4)	^∗^	^∗^	^∗^	^∗^	^∗^	^∗^
Hazardous (5–12)	26 (45.6)	34 (59.6)	1.76	0.84–3.74	0.135	**2.70**	**1.14–6.81**	**0.029**
Problematic substance use (DAST-10), *n* (%)								
No (0–2)	43 (75.4)	35 (61.4)	^∗^	^∗^	^∗^	NI	NI	NI
Yes (≥3)	14 (24.6)	22 (38.6)	1.93	0.87–4.39	0.109	NI	NI	NI
Sexual stigma sum score (0–33), median (IQR)	4 (0–11)	5 (1–9)	1.00	0.95–1.06	0.946			
HIV-stigma sum score (12–48), mean (SD)	25.2 (6.5)	28.1 (7.0)	1.07	1.01–1.13	0.027	NI	NI	NI

Results from the univariable logistic regressions. Variables with a *P* value ≤ 0.15 were preselected. The right three columns capture results of the final prediction model for the case-control study after multivariable logistic regression and stepwise backwards selection (*P* < 0.05). ART, antiretroviral therapy; AUDIT, alcohol use disorders identification test; GAD, generalized anxiety disorder; IQR, interquartile range; NI, not included in the final model; SD, standard deviation.

∗ Reference category.

The final prediction model after backwards stepwise selection included missed more than 4 days on ART per month (OR = 5.54, 95% CI 1.19–41.03, *P* = 0.048), anxiety (OR = 3.90, 95% CI 1.34–12.52, *P* = 0.016), hazardous alcohol use (OR = 2.70, 95% CI 1.14–6.81, *P* = 0.029), and Christian religion vs. no religion (OR = 3.89, 95% CI 1.34–12.29, *P* = 0.015), Table 2.).

## Discussion

Understanding factors associated with unsuppressed viral load among MSM with HIV in South Africa is crucial for national and international milestones for preventing HIV transmission. Our study identified several predictors of unsuppressed viral load that can be screened for at ART service enrolment or continuation service visits.

Findings from our cross-sectional analysis of POP INN data identify being an outreach client as an important predictor of unsuppressed viral load. Outreach clients face significant barriers to care, such as staff shortages, vehicle breakdowns, and difficulties locating clients. Consequently, some outreach clients may lack timely medication distribution, counselling and viral load monitoring, leading to higher unsuppressed viral load rates [28–30]. Improving outreach care coordination, possibly by involving community healthcare workers, family support, or buddy programs, may enhance healthcare ownership among these clients [31,32]. We also found that clients on ART less than 5 years were more likely to have unsuppressed viral load. Notably, young age was not a predictor of unsuppressed viral load in our study [33] due to the low proportion (4.9%) of young clients (aged 18–25 years) in our sample [8].

Findings from our case–control study identify missing more than four ART doses per month as significant predictor of unsuppressed viral load, confirming the link between nonadherence and poor viral load outcomes [9,34]. Clients with poor adherence could be identified through tools like the Visual Analogue Scale (VAS) during counseling appointments [35]. Moderate to severe anxiety was also identified as a predictor of unsuppressed viral load, consistent with previous research [36]. Although unemployment and HIV stigma-related fear of disclosure may have been related to anxiety, no direct association was found with unsuppressed viral load in our study. Hazardous drinking behavior was another predictor of unsuppressed viral load, aligning with prior studies linking alcohol use to nonadherence [37].

Early identification of clients with hazardous drinking behavior could help identify those with unsuppressed viral load. Although we did not find associations between other mental health challenges and unsuppressed viral load, more than half of the participants, both controls and cases, reported experiencing abuse or problematic substance use. The high prevalence of mental health challenges suggests that incorporating a brief mental health screening tool during client intake, along with routine assessments of ART adherence, could improve client-specific counseling and overall health outcomes in the POP INN clinics. However, there is a need to develop mental health interventions that are relevant and accessible to MSM with HIV in the South African context, as no current efficacious mental health interventions for this population are known to exist [13]. Last, our study identified Christian faith as a predictor of unsuppressed viral load. Religiosity is a growing area of interest in HIV research, and some findings suggest religion may be linked to misinformation about HIV or unconventional healing practices promoted by some faith-based organizations that discourage ART adherence [38].

The case–control study's limitations included a restricted sample size, which limits precision in identifying predictors. Self-reported measures and the use of a translator in some cases may have introduced recall bias and social desirability bias, potentially underestimating associations. A general limitation in our study is the inclusion of only active POP INN clients and the cross-sectional design. Larger studies are required to confirm these findings.

In conclusion, our study identified key predictors of unsuppressed viral load among MSM with HIV, including outreach clients, shorter ART duration, missed doses, anxiety, hazardous drinking, and Christian faith. Identifying and providing adherence counselling and other supportive interventions to individuals who are more likely to be experiencing unsuppressed viral load is necessary to advance UNAIDS 95-95-95 targets.

## Acknowledgements

The authors acknowledge the Centers for Disease Control and Prevention support to Aurum's POP INN program in South Africa.

Funding for the study was provided in part through the Sub-Saharan African Network for TB/HIV Research Excellence (SANTHE), which is funded by the Science for Africa Foundation to the Developing Excellence in Leadership, Training and Science in Africa (DELTAS Africa) program [Del-22-007] with support from Wellcome Trust and the UK Foreign, Commonwealth & Development Office and is part of the EDCPT2 program supported by the European Union; the Bill & Melinda Gates Foundation [INV-033558]; and Gilead Sciences Inc. [19275]. All content contained within is that of the authors and does not necessarily reflect positions or policies of any SANTHE funder. D.O. and E.J.S. were supported in part by NIH grant R34MH135806. All authors thank Dr Thomas H. Wieringa for his statistical consultation during the manuscript.

For the purpose of open access, the author has applied a CC BY public copyright license to any Author Accepted Manuscript version arising from this submission.

### Conflicts of interest

There are no conflicts of interest.

## Supplementary Material
