# Supplementary material for: Predictors of unsuppressed viral load among MSM with HIV in South Africa
Source: AIDS. 2025 May 21;39(9):1290–6. doi: 10.1097/QAD.0000000000004205 (PMC12237100; doi:10.1097/QAD.0000000000004205)
Supplement: Supplementary file 1 [file aids-39-1290-s001.docx]

# Supplemental material

**Appendix 1: Measures case-control study questionnaire**

- *ART adherence*: Attitudes towards taking ART, reasons missed, and disclosure status were asked in six subsequent questions, based on previous research in the clinics (1).
- *Abuse:* Abuse was assessed using the USAID HPI MSMS Trauma Screening Tool (2). This measure is specifically developed and validated for MSM and transgenders. It includes questions on four types of abuse: forced or coerced sex, physical abuse, emotional abuse, and threats or intimidation. All four questions have a yes or no answer. A positive response to any of the questions was interpreted as experienced abuse (2).
- *Depression:* The Patient Health Questionnaire (PHQ)-9 module is a validated measure to assess depression among African HIV-seropositive adults and MSM (3,4). It includes nine questions rated on a 4-point Likert scale (5). The summed score can range from 0 to 27 and scores $\geq$ 10 were interpreted as having depression.
- *Anxiety*: The Generalized Anxiety Disorder (GAD)-7 module is a validated measure to assess anxiety among high HIV prevalence populations in sub-Saharan Africa (6). It includes seven questions rated on a 4-point Likert scale (7). The summed score can range from 0 to 21 and scores $\geq$ 10 were defined as the presence of anxiety.
- *Alcohol abuse:* Alcohol abuse was measured using the widely used WHO Alcohol Use Disorder Identification Test (AUDIT)-C module (8). It has been validated among patients in HIV care in South Africa (9). It composes 3 questions with scores between 0-4. The summed score can range from 0-12 and scores $\geq$ 4 were interpreted as hazardous drinking behaviour.
- *Substance abuse*: Substance abuse other than alcohol and tobacco was measured using the validated Drug Abuse Screening Test (DAST)-10 module (10). A widely validated and used measure to assess drug use (11). All ten questions have a yes or no answer. Every ‘yes’ adds one point to the maximum score of 10, where a total score of $\geq$ 3 was interpreted as harmful substance abuse.
- *Sexual stigma*: Sexual stigma was assessed with the abbreviated version of the China MSM Stigma Scale (12). The module includes questions on both perceived and enacted stigma. The instrument has been adjusted for our research cohort: ‘homosexuality’ was replaced with ‘men who have sex with men’ to better align with the participants' circumstances (13). The eleven questions included answers on frequency of perception and were scored on a scale from 0-3 (never; once or twice; a few times; many times). The summed score can range from 0-33. A higher score indicated a greater perception of sexual stigma (14).
- *HIV stigma:* The 12-item short-version of the Berger HIV Stigma scale was used to assess HIV stigma (15). This measure has been validated in several sub-Saharan countries (16,17). Twelve questions are spread over four different domains: personalised stigma, disclosure concerns, concerns about public attitudes, and negative self-image. All questions are rated on a 4-point Likert scale and the summed score can range from 12 to 48. A higher score indicated a greater perception of HIV stigma (15).

**References**

1. Feldman BJ, Fredericksen RJ, Crane PK, Safren SA, Mugavero MJ, Willig JH, et al. **Evaluation of the single-item self-rating adherence scale for use in routine clinical care of people living with HIV**. *AIDS Behav*. 2013; **17**:307–18.

2. Betron M. **Screening for Violence against MSM and Transgenders: Report on a Pilot Project in Mexico and Thailand**. Washington, DC; 2009. https://scholar.google.com/citations?view_op=view_citation&hl=en&user=dxhRmV8AAAAJ&citation_for_view=dxhRmV8AAAAJ:MXK_kJrjxJIC

3. Cholera R, Gaynes BN, Pence BW, Bassett J, Qangule N, Macphail C, et al. **Validity of the Patient Health Questionnaire-9 to screen for depression in a high-HIV burden primary healthcare clinic in Johannesburg, South Africa**. *J Affect Disord* 2014; **167**:160–6.

4. Ulanja MB, Lyons C, Ketende S, Stahlman S, Diouf D, Kouamé A, et al. **The relationship between depression and sexual health service utilization among men who have sex with men (MSM) in Côte d’Ivoire, West Africa**. *BMC Int Health Hum Rights* 2019; **19**.

5. **Patient Health Questionnaire-9 (PHQ-9) - Mental Health Screening** - National HIV Curriculum.

6. Chibanda D, Verhey R, Gibson LJ, Munetsi E, Machando D, Rusakaniko S, et al. **Validation of screening tools for depression and anxiety disorders in a primary care population with high HIV prevalence in Zimbabwe**. *J Affect Disord*. 2016; **198**:50–5.

7. **Generalized Anxiety Disorder 7-item (GAD-7) - Mental Health Screening** - National HIV Curriculum.

8. Babor TF, Higgins-Biddle JC, Saunders JB, Monteiro MG. **The Alcohol Use Disorders Identification Test (AUDIT) manual: Guidelines for use in primary care**. 2001.

9. Morojele NK, Nkosi S, Kekwaletswe CT, Shuper PA, Manda SO, Myers B, et al. **Utility of Brief Versions of the Alcohol Use Disorders Identification Test (AUDIT) to Identify Excessive Drinking Among Patients in HIV Care in South Africa**. *J Stud Alcohol Drugs* 2017; **78**:88–96.

10. Skinner HA. **The drug abuse screening test**. *Addictive behaviors* 1982; **7**:363–71.

11. Yudko E, Lozhkina O, Fouts A. **A comprehensive review of the psychometric properties of the Drug Abuse Screening Test**. *J Subst Abuse Treat* 2007; **32**:189–98.

12. Logie CH, Newman PA, Chakrapani V, Shunmugam M. **Adapting the minority stress model: associations between gender non-conformity stigma, HIV-related stigma and depression among men who have sex with men in South India**. *Soc Sci Med.* 2012; **74**:1261–8.

13. Secor AM, Wahome E, Micheni M, Rao D, Simoni JM, Sanders EJ, et al. **Depression, substance abuse and stigma among men who have sex with men in coastal Kenya**. *AIDS* 2015; **29**:S251.

14. Korhonen CJ, Flaherty BP, Wahome E, Macharia P, Musyoki H, Battacharjee P, et al. **Validity and reliability of the Neilands sexual stigma scale among Kenyan gay, bisexual, and other men who have sex with men**. *BMC Public Health* 2022; **22**:1–10.

15. Reinius M, Wettergren L, Wiklander M, Svedhem V, Ekström AM, Eriksson LE. **Development of a 12-item short version of the HIV stigma scale**. *Health Qual Life Outcomes* 2017; **15**:1–9.

16. Wanjala SW, Nyongesa MK, Mwangi P, Mutua AM, Luchters S, Newton CRJC, et al. **Measurement characteristics and correlates of HIV-related stigma among adults living with HIV: a cross-sectional study from coastal Kenya**. *BMJ Open* 2022; **12**:050709.

17. Alemu A, Meskele M, Darebo TD, Handiso TB, Abebe A, Paulos K. Perceived HIV **Stigma and Associated Factors Among Adult ART Patients in Wolaita Zone, Southern Ethiopia***. HIV/AIDS - Research and Palliative Care* 2022; **14**:487–501.
